# Visual perceptual load and processing of somatosensory stimuli in primary and secondary somatosensory cortices

**DOI:** 10.1038/s41598-023-34225-5

**Published:** 2023-04-28

**Authors:** Antje Peters, Laura Brockhoff, Maximilian Bruchmann, Torge Dellert, Robert Moeck, Insa Schlossmacher, Thomas Straube

**Affiliations:** 1 Institute of Medical Psychology and Systems Neuroscience, University Hospital Münster, Von-Esmarch-Straße 52, 48149 Münster, Germany; 2 Otto Creutzfeldt Center for Cognitive and Behavioral Neuroscience, University of Münster, 48149 Münster, Germany

**Keywords:** Attention, Perception, Sensory processing, Neuroscience, Somatosensory system, Cortex

## Abstract

*Load theory* assumes that neural activation to distractors in early sensory cortices is modulated by the perceptual load of a main task, regardless of whether task and distractor share the same sensory modality or not. While several studies have investigated the question of load effects on distractor processing in early sensory areas, there is no functional magnetic resonance imaging (fMRI) study regarding load effects on somatosensory stimuli. Here, we used fMRI to investigate effects of visual perceptual load on neural responses to somatosensory stimuli applied to the wrist in a study with 44 participants. Perceptual load was manipulated by an established sustained visual detection task, which avoided simultaneous target and distractor presentations. Load was operationalized by detection difficulty of subtle or clear color changes of one of 12 rotating dots. While all somatosensory stimuli led to activation in somatosensory areas SI and SII, we found no statistically significant difference in brain activation to these stimuli under high compared to low sustained visual load. Moreover, exploratory Bayesian analyses supported the absence of differences. Thus, our findings suggest a resistance of somatosensory processing to at least some forms of visual perceptual load, possibly due to behavioural relevance of discrete somatosensory stimuli and separable attentional resources for the somatosensory and visual modality.

## Introduction

*Load Theory*^1–5^ is the dominant theory of attentional influences on information processing. It proposes that the extent of processing of task-irrelevant stimuli depends on the level and type of task load. In the case of low perceptual load, distractor-stimuli are processed since enough attentional resources are available. In contrast, under high perceptual load, the capacity of the perceptual system might be exhausted, leaving no room for processing irrelevant stimuli. Load theory suggests that, specifically, the high load condition leads to early effects on distractor processing, while attentional effects are seen at later stages under low-load conditions^1–5^. Remarkably, the theory makes no assumptions about whether task load and distractor have to be from the same modality^5^. In several experiments of the proponents of this theory, the load condition was realized in another modality than the distractor modality (e.g. Refs.^6,7^). In neuroscientific terms, the supposed early suppressive effects of load on distractor activity should be seen in sensory areas corresponding to early processing areas in the cortical hierarchy^1–5^.

While several neuroscientific studies support load theory, other studies failed to find early effects, and, in general, load effects are more reliably seen during later processing stages (for review see Ref.^5^). Furthermore, the question of whether load effects are more pronounced in uni- than in crossmodal designs remains a matter of debate^5^. Classical theories have either proposed separate cognitive resources for different modalities^8^ or a single resource to which these modalities have access^9^. More recent approaches suggest that attention across modalities shares common resources but can also be separated for single modalities^10^. According to the broad formulation of Load theory, load effects should also be present in crossmodal experiments, in which irrelevant stimuli and load-inducing stimuli are from different sensory modalities. Thus, the load of a given task should block or at least reduce activation in early sensory areas^3,5^. However, even though at least late load effects are consistently shown in both unimodal and multimodal studies^5^, it has been suggested that differences between uni- and multimodal designs may exist, leading to stronger effects in unimodal designs (for discussion see Ref.^5^). Specifically, in unimodal designs, perceptual load might be partially due to biased competition and lateral inhibition in early sensory areas (e.g. Ref.^11^).

In accordance with *Load Theory*, several functional magnetic resonance imaging (fMRI) studies showed that perceptual load affects activation in sensory areas to visual and auditory distractor stimuli in unimodal (e.g. Refs.^12–17^) and crossmodal designs (e.g. Refs.^18,19^). Similarly, studies with event-related potentials (ERPs) showed load effects on early visual or auditory distractor processing in both uni- (e.g. Refs.^20–25^) and crossmodal designs (e.g. Refs.^6,26,27^). However, other studies did not report early effects in fMRI or electroencephalographic studies^5^. A recent review concluded that load manipulations are more consistently seen during late processing stages, even though it is often difficult to separate effects on distractors from target-related activity^5^.

Remarkably, to our knowledge, no fMRI study investigated whether perceptual load affects neural responses to tactile distractors in primary and secondary somatosensory cortices. The primary somatosensory cortex (SI) is structured somatotopically^28^. It receives information from somatosensory nerve fibers, which originate in the contralateral body half^29,30^. The afferent pathways cross to the opposite side on the way to the thalamus in the spinal cord or brainstem^31^. In contrast to SI, the secondary somatosensory cortex (SII) represents higher levels of processing of somatosensory stimuli. It has great importance for the interpretation and categorization of somatosensory information^32^. Lesions of SII result in tactile agnosia^33^. SII is less lateralized than SI and represents both body halves on each side. Nevertheless, the SII of the non-dominant hemisphere is essential for spatial orientation^34^. Moreover, SII is found to perform higher cognitive tasks, such as the integration of different modalities and self-consciousness^35^. The hierarchical organization of the somatosensory system is also supported by monkey studies, with initially strictly contralateral processing followed by intrahemispheric transfer leading to bilateral processing of somatosensory stimuli in postcentral associative somatosensory cortex and secondary somatosensory cortex^29,30,35,36^.

While there are no fMRI studies on the effects of perceptual load on the processing of somatosensory distractors, several fMRI studies examined the effect of directing attention towards or away from a somatosensory stimulus on activity in SI and SII (e.g. Refs.^37–43^). While some studies found effects in SI and SII (e.g. Refs.^37,40–42^), other studies reported attention-dependent modulations only in SI^43^, only in SII^38^, or neither in SI nor in SII^39^. Findings might vary with specific experimental parameters such as stimulus type and location. Furthermore, an additional factor might be the perceptual or also the general load during a task that directs attention away from the distractors. In this vein, there is one study^44^ that investigated the effects of a visual working memory load manipulation on brain responses to tactile stimuli. This study showed no effect of working memory load on tactile stimulation in SI or SII.

Furthermore, while there are no fMRI studies on perceptual load effects on tactile stimuli, processing of somatosensory stimuli during visual tasks has been studied in electroencephalography (EEG) studies^19,45–48^. Thus, it has been shown, for example, that a secondary visual task reduces early ERPs to tactile stimuli compared to a single task situation^48^. Other studies found that attention to body-related visual information or the amount of a working memory task of hand images enhances somatosensory ERPs^45–47^. This suggests a supramodal model of attention. However, it leaves the question of whether the perceptual load of a visual task modulates early somatosensory ERPs to task-unrelated somatosensory distractors. This has been investigated in one study, which found effects of visual task load on latencies but not amplitudes of somatosensory ERPs^19^. Behavioural studies suggest that both visual^49^ and tactile^50^ load conditions can induce inattentional numbness for singular and unexpected somatosensory stimuli. However, it is unclear whether this represents an early effect in neuronal activity and whether numbness can be seen in designs with multiple presentations of tactile distractors.

Taken together, it remains to be answered whether load-dependent modulations of somatosensory cortices during distractor processing can be shown in suited fMRI or other neuroscientific studies. Load effects on somatosensory distractor processing in sensory areas would be in accordance with load theory, which proposes a suppression of early activation under high load. Due to the unspecific assumptions of load theory, effects should be found regardless of the modality of the main task, similar to studies that used crossmodal designs to show early suppression of distractor-related brain responses in the auditory or visual modality^18,19^. Based on these considerations, the current study aimed to investigate the effects of perceptual load on the processing of somatosensory stimuli in primary and secondary sensory areas. We hypothesized that effects of perceptual load would lead to reduced activation in the somatosensory cortices. To test this hypothesis, we used a crossmodal design with manipulation of visual load. The used visual search task has previously been found to reduce early brain responses to visual distractors under the high- as compared to the low-load condition^20^. The goal of the present experimental paradigm was to generate perceptual load in an established and controllable way^5^ while avoiding the concurrent presence of target and distractor stimuli. This reduces the interpretative problem, whether effects are based on perceptual load or executive demands during decision-making.

Furthermore, if targets and distractors are presented simultaneously it becomes difficult to separate neural activation that is related to targets from activation related to distractors^5^. Applying a sustained visual search task and somatosensory stimuli made is easier to dissociate effects on distractors from task/target effects in the analysis of neuronal activation due to different modalities of task and distractor and the temporal separation of target and distractor trials^5^. Perceptual load of the visual search task was varied by the difficulty of perceptual discrimination^51^. We specifically investigated how this manipulation modulated activation in SI and SII to somatosensory stimuli since we considered these areas as early areas that should be affected by visual load according to Load Theory.

## Methods

### Subjects

The sample consisted of 47 participants recruited from the local student community of the University of Münster via public advertisements. Previous fMRI studies on attentional modulation of somatosensory processing have reported effect sizes between null and very large effects (e.g. Refs.^37,44^). We, therefore, assumed a medium effect size (*d* = 0.5) for a paired t-test (α = 0.05, β = 0.95). Power calculations with G*Power 3.1.9^52^ resulted in a required sample size of 45. Two additional participants were recorded due to expected dropouts. All participants had normal or corrected-to-normal visual acuity and no history of psychiatric or neurologic illness. Participants provided written informed consent before the experiment and received monetary compensation (10€/h). All procedures were approved by the ethics committee of the University of Münster and were conducted in accordance with the Declaration of Helsinki. Three participants could not be included in the analysis due to technical failures during the experiment. The final sample comprised 44 participants (33 female, 11 male) with a mean age of 24.4 years (SD = 3.6 years) and a range of 20–35 years.

### Stimuli and experimental paradigm

#### Somatosensory stimuli

Somatosensory stimuli were applied via the BIOPAC MP150 system with the stimulation module STM100C using the software AcqKnowledge 5.0 (BIOPAC Systems, Inc.). Two Ag/AgCl skin electrodes were attached to the participants' wrists to stimulate the left nervus radialis superficialis. The left fovea radialis was considered an anatomical landmark to obtain equal stimulation conditions. Via the skin electrodes, a clearly perceptible electric pulse burst (10 ms, consisting of 5 pulses of 2 ms) served as the distractor stimulus. Individual somatosensory thresholds for each participant were identified by gradually increasing and decreasing the voltage output of the BIOPAC stimulation module and asking subjects to report when they started or stopped to perceive the stimulation until the perception threshold voltage V_c_ was identified. The maximum voltage was 40 V, corresponding to a current of approximately 40–0.04 mA for a typical skin resistance of 1–100 kΩ^53^. Currents used for the remainder of the experiment corresponded to a voltage of 1.75 times the V_c_ to ensure clear above-threshold stimulation. Visual stimuli. At all times during the experiment, a white fixation cross of 0.9 × 0.9 degrees of visual angle (°) was presented centrally. Concurrently, 12 small red (RGB = [102, 0, 0]) dots rotated around the fixation cross on three concentric trajectories with radii of 0.7°, 1.3°, and 1.9° with a constant angular velocity of 60°/s. The radii of the dots per trajectory were 0.9°, 0.12°, and 1.15°. The rotation direction changed after 8 to 16 stimulus presentations. Occasionally, one of the dots changed its color for 200 ms, serving as a target for the visual task. The discriminability of the color change relative to the other dots varied between low and high discriminability (representing low and high load; see below) based on a previous study^20^. In this previous study, the load of the continuous visual search task modulated early and late responses to visual distractors. Similar versions of the visual search task have been used in several studies of our group to induce inattentional blindness^54,55^ or to investigate load effects on visual processing^56^. We used this crossmodal sustained visual task since it allowed dissociating the somatosensory distractor processing from the visual load modality and the responses to visual targets^5,20^. Participants were asked to react to the color change via keyboard presses with the right hand. Participants were instructed to keep their gaze at the fixation cross. The presentation was implemented in MATLAB and the Psychophysics Toolbox^57,58^. The image was projected onto a semitransparent screen positioned at the head end of the scanner with a resolution of 1920 × 1080 pixels at 120 Hz. Participants viewed the screen in a mirror attached to the head coil.

#### Experimental procedure

**Figure 1:**
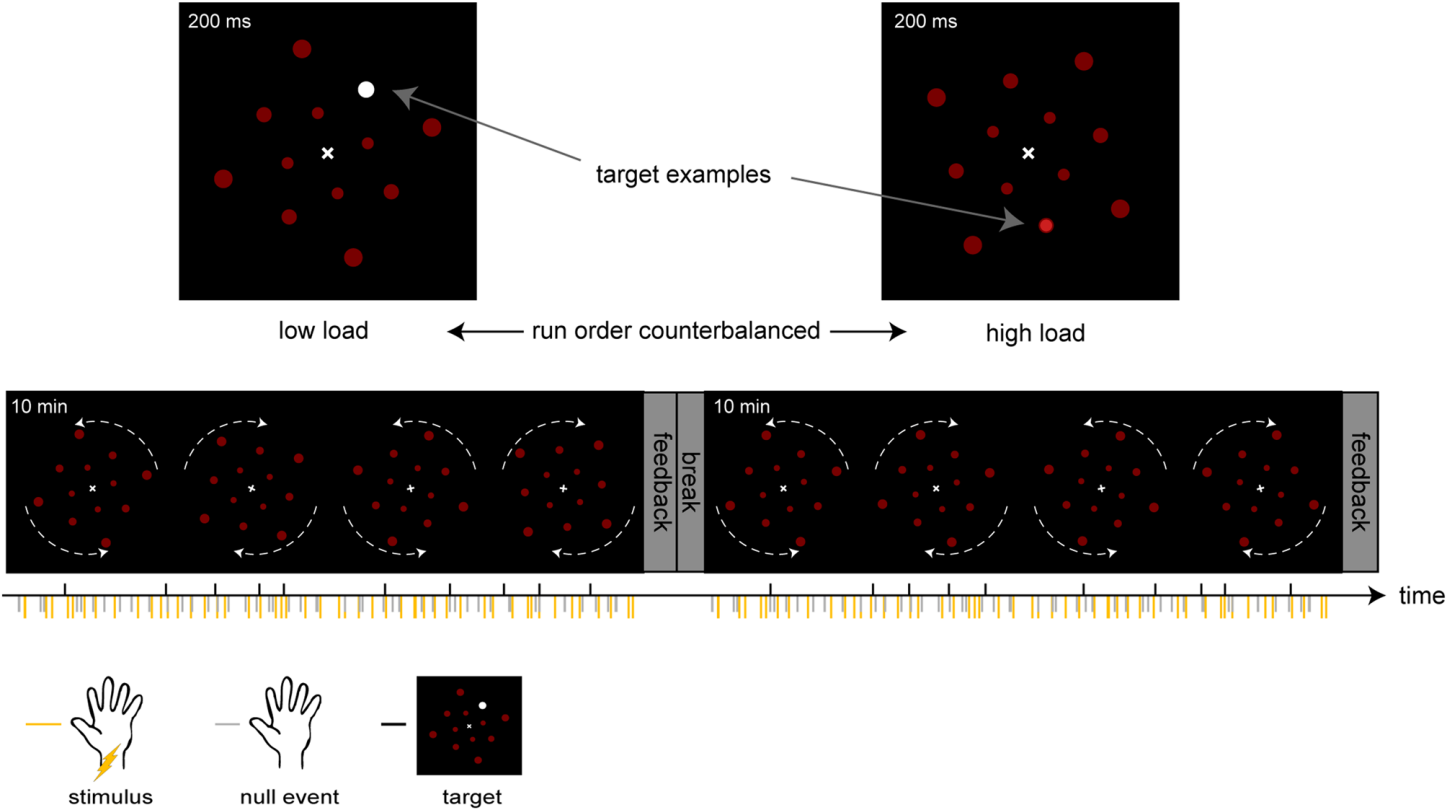
Schematic diagram of the experimental procedure. There was one low-load and one high-load run with 50 somatosensory stimulus events and 50 null events each. The high- and low-load conditions differed in the difficulty of the tasks: The target detection with subtle color change corresponded to the high-load task, and the detection with clearly visible color change corresponded to the low-load task. There were 10 target presentations per run. The order of the runs was counterbalanced between subjects. Rotation changes are indicated with dashed white arrows for illustration purposes. Their actual occurrence was more frequent and variable in timing (see main text for details). The timeline illustrates the time points of stimulus events (orange dashes), null events (grey dashes), and visual target stimuli (black dashes).

The main experiment consisted of two counterbalanced runs, one high-load, and one low-load run, with 50 presentations of somatosensory stimuli in each run. Additionally, 50 baseline events without somatosensory stimulation (“null events”) were defined per run. Low and high-load runs were counterbalanced across subjects. The order of stimulus and null events and the interstimulus interval (ITI) was determined by the Optseq algorithm (http://www.surfer.nmr.mgh.harvard.edu/optseq/)^59^. The Optseq algorithm generates a series of stimulus schedules in which the order of stimulus and null events and the distribution of inter-stimulus intervals is optimized to achieve maximal statistical sensitivity during first-level analysis (see below) within user-defined restrictions. In our case, these restrictions pertained to the number of stimuli per run (50 stimuli and 50 null events) and the total run duration (10 min). Two of these schedules were used for all runs. The order of schedules and their assignment to either the low or high load run was counterbalanced across subjects. In both schedules, the resulting ITIs had a mean of 6 s (minimum of 1.8 s, maximum of 10.8 s). Visual target stimuli were displayed 10 times per run session (on average, every 60 s). They occurred between 10 randomly chosen pairs of stimulus (or null) events in the middle of the ITI. During the low-load run, a target stimulus was defined by a color change of a randomly selected dot for 200 ms from red to white (RGB = [255, 255, 255]). During the high-load task, dots changed their color from red to bright red (RGB = [204, 26, 26]). No feedback was given during experimental runs, but participants were provided with the percentage of correct reactions during the visual task at the end of each run to encourage engagement. Runs lasted 10 min each, and participants were allowed to take breaks between runs when needed. Figure 1 shows a schematic diagram of the experimental structure.

### Behavioural data analysis

Task performance was quantified by each subject's response times (RTs) and hit rate. Paired two-tailed t-tests (α = 0.05) were used to compare the performance parameters of all subjects between the high- and low-load conditions. In the case of a 0% hit rate during the high-load condition, no response time could be recorded. This was the case for 5 participants.

### Image acquisition and analysis

*FMRI data acquisition*. A 3-Tesla Siemens Magnetom Prisma and a 20-channel Siemens Head Matrix Coil (Siemens Medical Systems) were used for fMRI data collection. We recorded a high-resolution T1-weighted scan with 192 slices with a repetition time (TR) of 2130 ms, an echo time (TE) of 2.28 ms, a flip angle (FA) of 8°, and a voxel size of 1 × 1 × 1 mm within a field of view (FOV) of 256 × 256 mm. During the tasks, two datasets were acquired per subject with a T2*-weighted echoplanar sequence sensitive to blood oxygenation level-dependent (BOLD) contrast (TR = 2300 ms, TE = 30 ms, FA = 90°, FOV = 216 × 216 mm, voxel size = 3 × 3 × 3 mm). Datasets comprised 274 volumes, each with 42 interleaved axial slices (thickness = 3 mm, gap = 0.3 mm) orientated in an approximately 25° tilted angle from the anterior–posterior commissure plane in order to reduce susceptibility artifacts in inferior parts of anterior brain areas. Before functional imaging, a shimming field was applied to minimize magnetic field inhomogeneity.* fMRI data preprocessing*. We used MATLAB 9.7 (MathWorks) with SPM12 version 7771 (The Wellcome Centre for Human Neuroimaging, UCL Queen Square Institute of Neurology, London, UK; https://www.fil.ion.ucl.ac.uk/spm/software/spm12/) and the Data Processing & Analysis of Brain Imaging (DPABI) 6.0 toolbox^60^ for preprocessing. To account for spin saturation effects, the first five data volumes were discarded. We applied slice time correction to the remaining volumes. Next, volumes were realigned using a six-parameter (rigid body) linear transformation, and the anatomic and functional images were coregistered. We used DARTEL^61^ for nonlinear spatial normalization of the data to Montreal Neurological Institute (MNI) standard space. Finally, data were spatially smoothed with a 6 mm full-width at half-maximum Gaussian kernel.

#### fMRI data analysis

**Figure 2:**
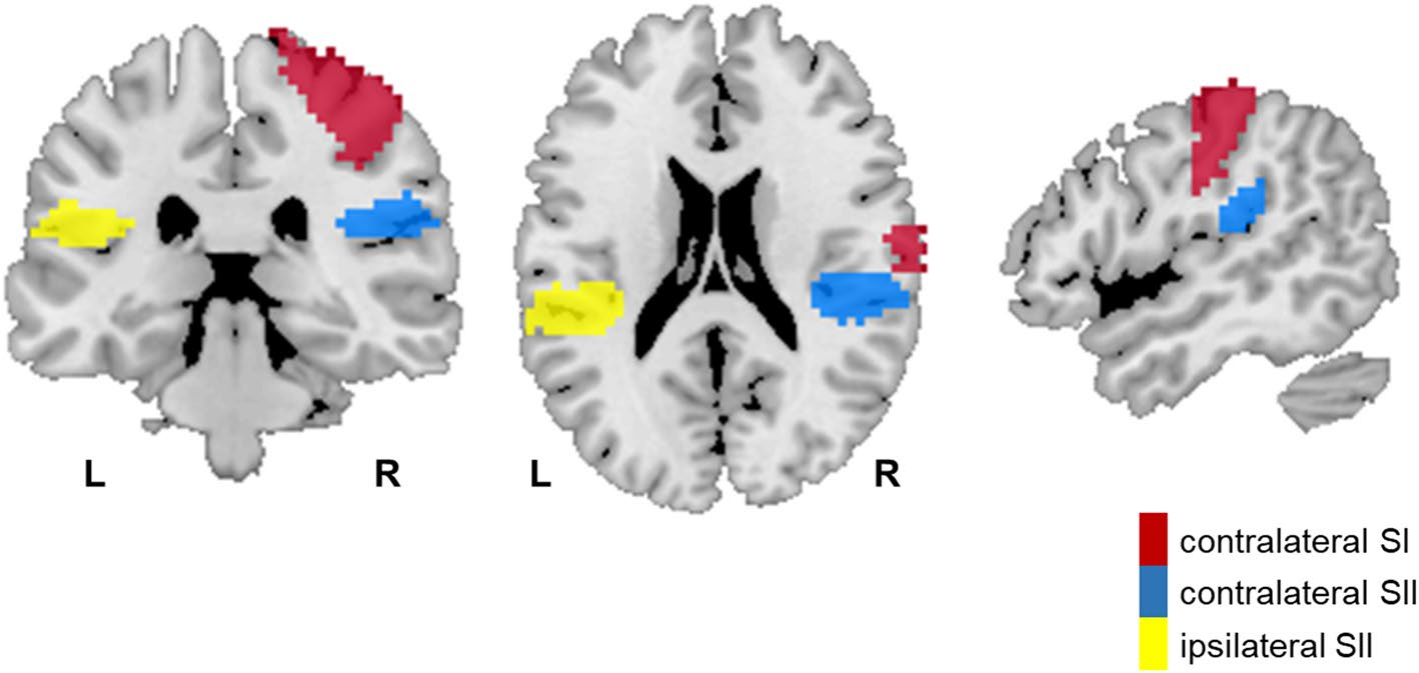
Regions of interest where processing of somatosensory stimuli was investigated: The primary somatosensory cortex contralateral to the stimulus application SI (red) and the secondary somatosensory cortex in both hemispheres SII contralateral (blue) and SII ipsilateral (yellow). Displayed layers intersect at x, y, z = 50, − 30, 20.

Data were cleared of slow signal drifts through a high-pass filter with a cutoff of 128 s. To model autocorrelations, we used the SPM pre-whitening method FAST^61,62^. To investigate the effects of the somatosensory stimulation, we performed first-level analyses of the data using a general linear model (GLM) for each participant. The GLM design matrix included four predictors of interest: somatosensory stimuli under high and under low load and null events without somatosensory stimulation under high and under low load, respectively. The visual target presentation under high and under low load, the time point of the participants' responses, and six head movement parameters were defined as predictors of no interest. These onsets were convolved with a 2-gamma hemodynamic response function to model the BOLD signal change for each predictor. Based on the first-level analysis, we computed different contrast images of the β-estimates for each participant: 1) the stimulus effect across load conditions (stimulus–no stimulus) and 2) the stimulus × load interaction (low load (stimulus–no stimulus)-high load (stimulus–no stimulus)). In a second-level analysis, we identified significant clusters across the whole group related to the stimulus effect and the stimulus × load interaction in our regions of interest (ROIs). The ROIs, which included the right primary somatosensory cortex (gyrus posterior, SI) and the left and right secondary somatosensory cortex (parietal operculum, SII^63^), were identified based on the Harvard Oxford Cortical Structural Atlas with 3 mm resolution, cf. Fig. 2. Another part of our analysis focused on the neural load effect, for which we performed first-level analyses with a different GLM. This time, we only included participants who gained more than 5 hits in the visual task for the target effects to analyze a sufficient number of trials per participant. The design matrix included six predictors of interest: somatosensory stimuli under high and under low load, null events without somatosensory stimulation under high and under low load, and target hit trials under high and low load. The visual target presentation associated with misses under high and under low load, and six head movement parameters were defined as predictors of no interest. The onsets were convolved with a 2-gamma hemodynamic response function to model the BOLD signal change for each predictor. Based on the first-level analysis, we computed contrast images of the β-estimates for each participant with the contrast: load effects during target processing ((high-load hits–no stimulus)-(low-load hits–no stimulus)). In a second-level analysis, we identified significant clusters across the whole group related to these contrasts in our regions of interest (ROIs). As a manipulation check, target effects were investigated in a task-difficulty ROI defined as in Ref.^64^, including parts of the insular cortices, the anterior cingulate cortex, the medial and lateral prefrontal cortex, and the intraparietal sulcus.

Furthermore, we also investigated activation in visual ROIs (cf., Harvard Oxford Cortical Structural Atlas with 3 mm resolution and Yeo atlas^65^). Please note that these analyses are only provided for transparency. This was not our main aim, and analyses in visual areas are difficult to interpret due to perceptual differences in the load conditions during target trials.

For each of the contrasts and each ROI, we performed cluster-based permutation tests with a voxel-level-threshold of α_voxel_ = 0.001, 5000 permutations, and cluster-mass statistics using PALM^66^. The p-values were FWER-corrected^67^. Only clusters that passed the cluster threshold α_cluster_ < 0.05 were considered significant.

## Results

### Behavioral data

As expected, the mean task reaction time was increased in the high-load (mean = 657 ms; SD = 375 ms) compared to the low-load condition (mean = 490 ms; SD = 65 ms; *t(38)* = − 2.76; *p* = 0.009; Cohen’s *d* = − 0.62) and hit rates were lower in the high- (mean = 57.73%; SD = 36.82%) compared to the low-load condition (mean = 89.55%; SD = 27.62%; *t(43)* = 6.48; *p* < 0.001; Cohen’s *d* = 0.98).

### Functional MRI data

#### Brain responses to visual targets in task-difficulty ROIs and occipital cortex depending on the load condition

**Figure 3:**
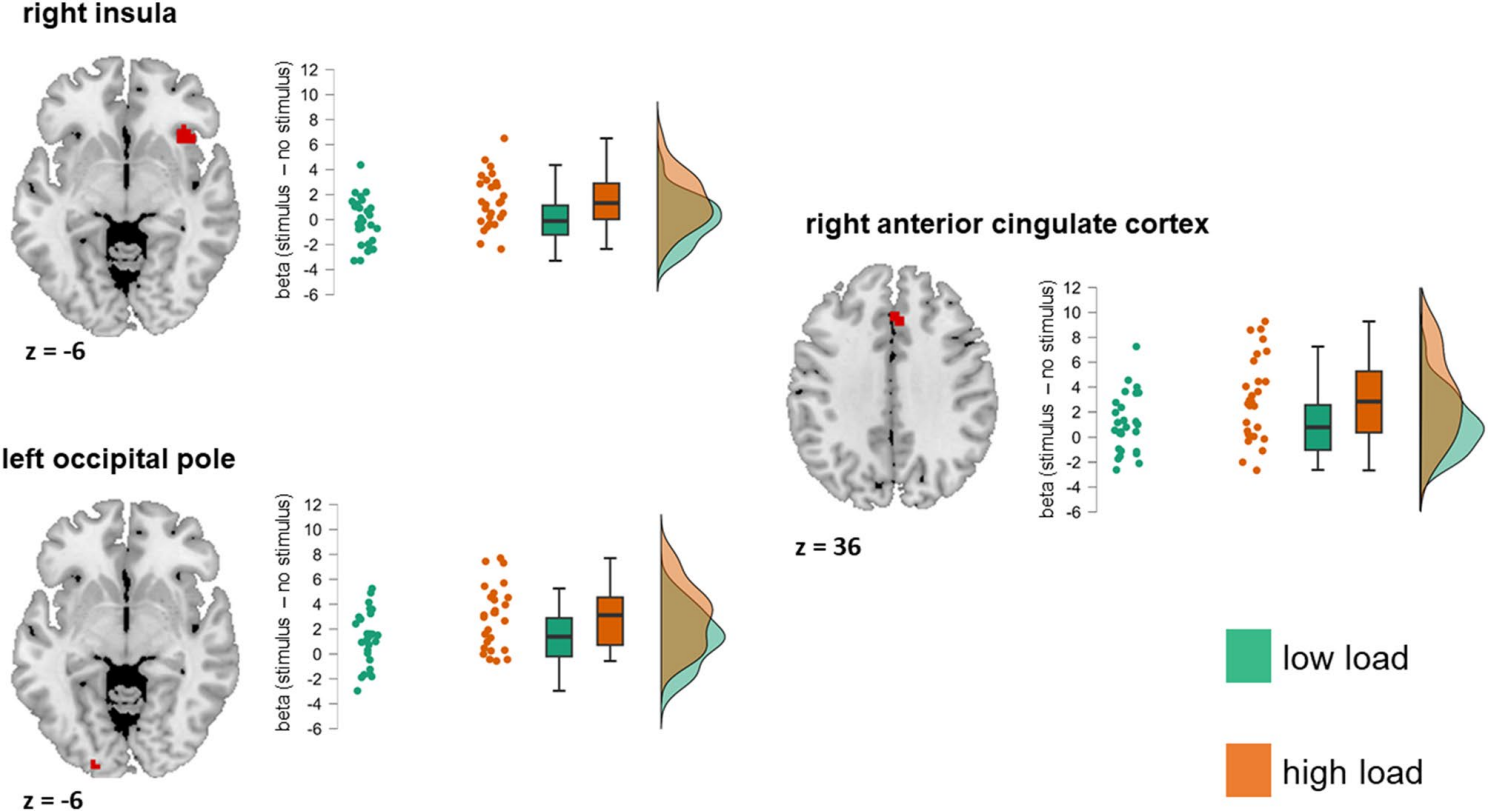
Clusters of increased activity in the load contrast during target processing in the task-difficulty ROIs and visual areas for N = 27 participants. In the task-difficulty ROIs, increased activation was found under high load in the right insula (peak *t*-value = 5.40, *p* < 0.05) and the right anterior cingulate cortex (peak *t*-value = 4.09, *p* < 0.05). In visual areas, one cluster of increased activity under high load was observed in the left occipital pole (peak *t*-value = 4.23, *p* < 0.05). Displays of the clusters are accompanied by raincloud plots of the individual data points in each condition, comprising a jittered scatter plot of the cluster average of the betas per subject, a box-and-whisker plot, and a violin plot of the same data.

We included participants with at least 6 hits in the visual task (N = 27). We found significantly increased activation in hits under high load compared to hits under low load in the right insula (peak *t*-value = 5.40, x, y, z = 36, 24, − 6; cluster size: 22 voxels) and in the right anterior cingulate cortex (peak *t*-value = 4.09; x, y, z = 3, 27, 36; cluster size: 18 voxels). In visual areas, we detected one cluster in the occipital pole (peak *t*-value = 4.23, x, y, z = − 21, − 96, − 6; cluster size: 5 voxels). See Fig. 3 for all target effects.

#### Brain activation in somatosensory cortex during distractor processing

We found significantly increased activation in the stimulus compared to the no stimulus condition across load conditions in the contralateral SI in two clusters: cluster 1 in BA 4 (peak *t*-value = 3.59, x, y, z = 42, − 33, 66; cluster size: 7 voxels) and cluster 2 in BA 48 (peak *t*-value = 4.64; x, y, z = 57, − 18, 24; cluster size: 8 voxels). Cluster 1 represents activation in the hand area^68^, while cluster 2 in the operculum might partially overlap with SII activations.

Furthermore, we found one cluster of significant activation for the main stimulation effect in the ipsilateral (i.e. left) SII (peak *t*-value = 4.96, x, y, z = − 57, − 24, 18; cluster size: 53 voxels) and one cluster in the contralateral SII (peak *t*-value = 6.03; x, y, z = 42, − 21, 18; cluster size: 132 voxels). Significant clusters are visualized in 4, including the beta values depending on the load condition.

**Figure 4:**
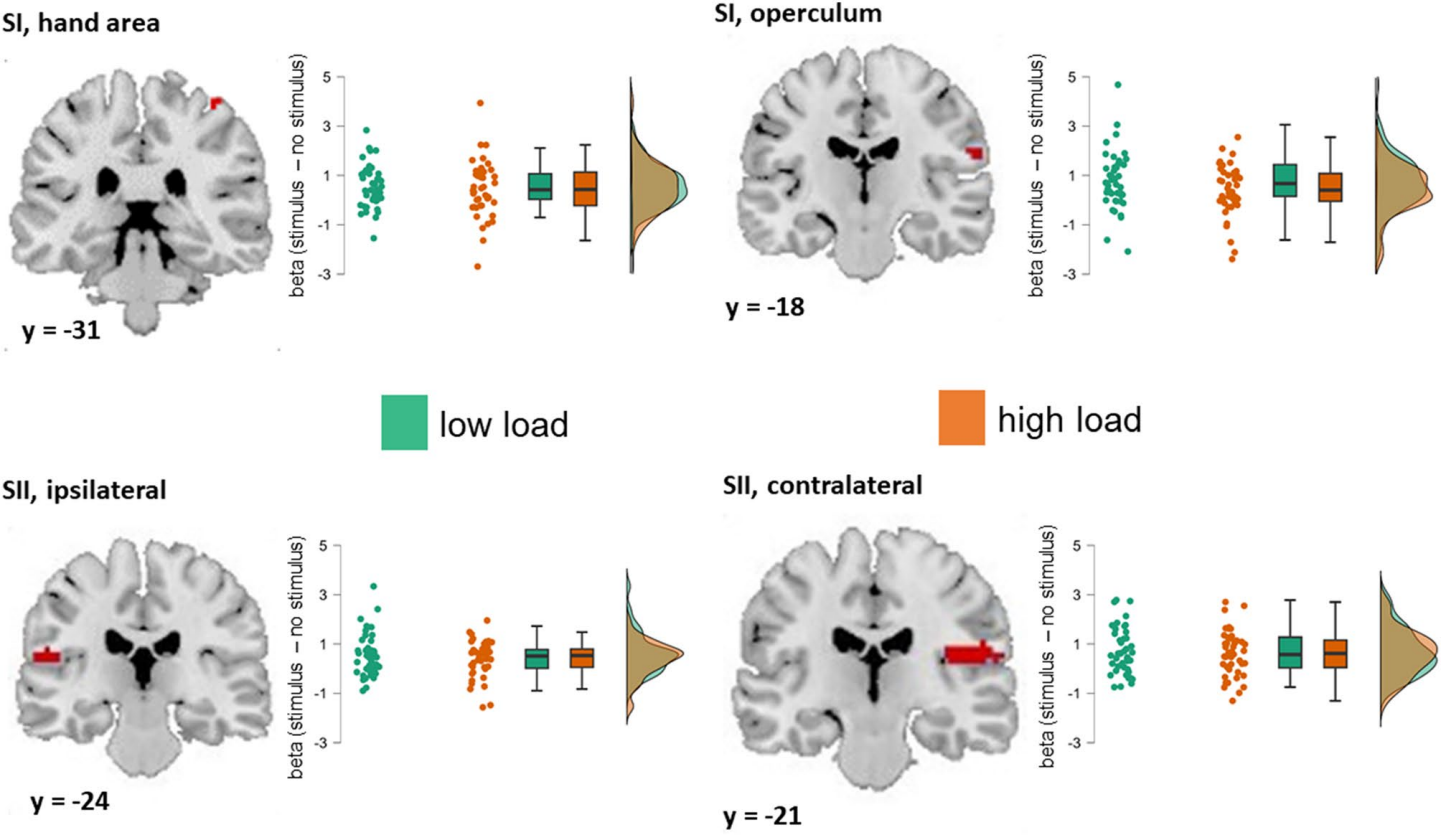
Clusters of increased activity in the stimulus compared to the no stimulus condition in the somatosensory areas in the primary and secondary somatosensory cortices (displayed in Fig. 2) for N = 44 participants. In the primary somatosensory cortex, three clusters with increased activity can be found: Two corresponding to the hand area of the left wrist, where the somatosensory stimuli were applied and one in the right parietal operculum in the neighbourhood of the secondary somatosensory cortex (cluster 1: peak *t*-value = 3.59, cluster 2: peak *t*-value = 4.64, both *p* < 0.05). In the secondary somatosensory cortex, one cluster can be found on each side (ipsilateral: peak *t*-value = 4.96, contralateral: peak t-value = 6.03, both *p* < 0.05). Displays of the clusters are accompanied by raincloud plots of the individual data points in each condition, comprising a jittered scatter plot of the cluster average of the betas per subject, a box-and-whisker plot, and a violin plot of the same data.

For the stimulus × load interaction, we found no statistically significant clusters in somosensory cortex ROIs (see Fig. 4). Furthermore, Bayes factors for the mean beta differences between load conditions based on the data shown in 4 suggest anecdotal to moderate evidence for the null hypothesis: BF_01_: low load (stimulus–no stimulus) = high load (stimulus–no stimulus))^69^; BF_01_(SI, hand area) = 3.57, BF_01_(SI, operculum) = 1.72, BF_01_(SII, ipsilateral) = 3.33 and BF_01_ (SII, contralateral) = 5.26. Thus, these analyses support the notion of the absence of differences between load conditions.

We additionally explored whether findings are modulated by interindividual differences in task performance, which might be associated with different sensitivity regarding distractor processing (e.g. Ref.^70^). Therefore, we correlated the beta-difference stimulus vs. no stimulus condition under high or low load with the continuous accuracy scores per load condition. There was no significant correlation (all *p* > 0.05).

**Figure 5:**
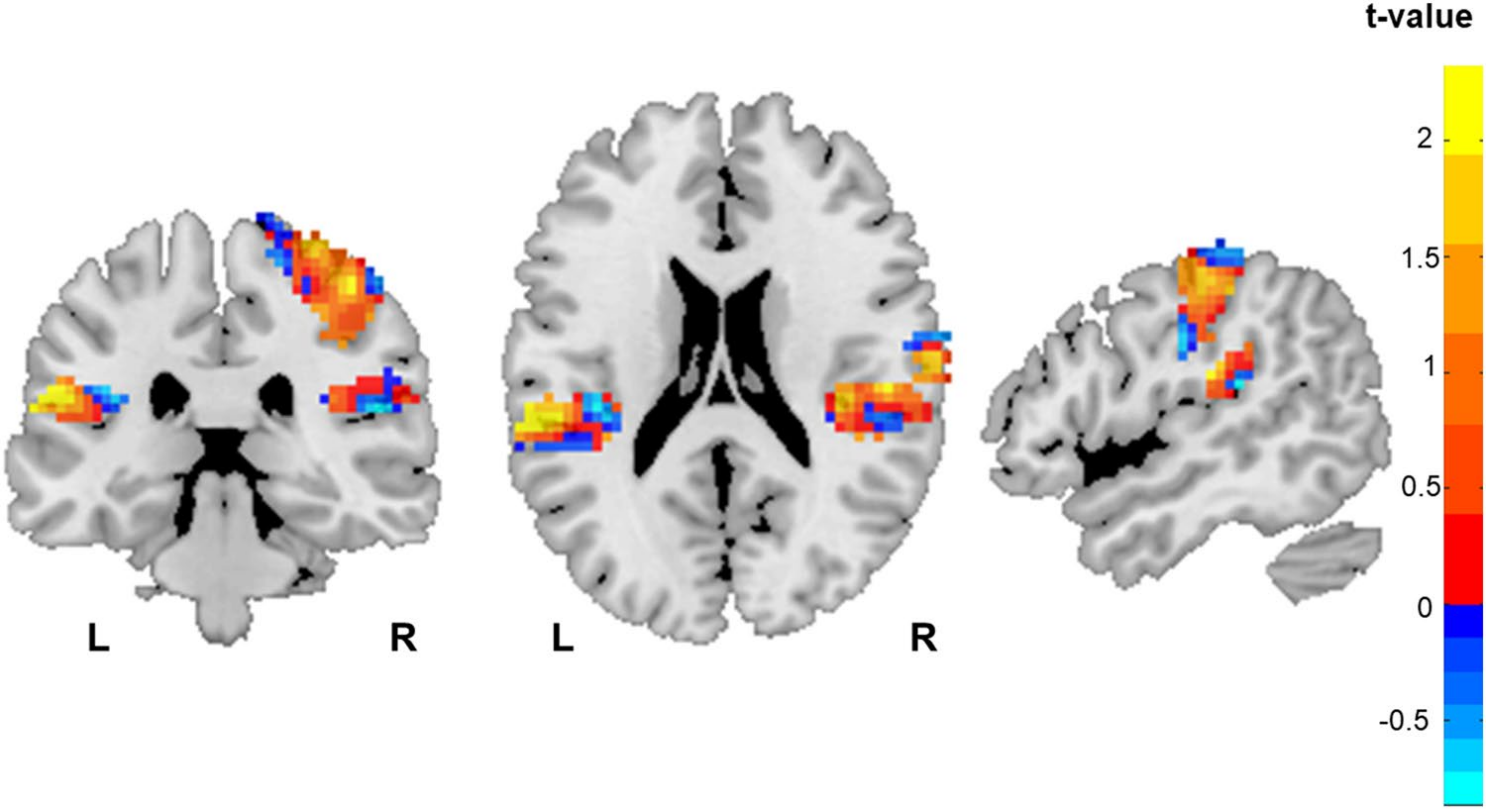
Voxelwise t-maps for the stimulus × load interaction in somatosensory areas (contralateral SI, contra-, and ipsilateral SII) for exploratory reasons. In this figure, the untresholded t-values per voxel for the contrast (low load (stimulus–no stimulus)—high load (stimulus–no stimulus)) are shown. While descriptively increased t-values are seen, please note that the permutation analysis did not reveal significant effects and that Bayes factors for the averaged activity in somatosensory areas support the null hypothesis.

Furthermore, for exploratory purposes serving the subsequent discussion of findings and the information of interested readers, we present un-thresholded t-maps for the contrast somatosensory activation under low vs high load in Fig. 5.

## Discussion

The aim of this study was to investigate whether perceptual load modulates neural responses to somatosensory stimuli in somatosensory areas. Despite a large sample size and in contrast to our hypothesis, we found no significant effect of visual load on neural responses to tactile distractors, and Bayesian analysis supported this null finding.

To our knowledge, our study was the first fMRI study to investigate the effects of perceptual load on the neural processing of somatosensory stimuli. Our findings partially contrast fMRI studies investigating perceptual load effects on visual or auditory stimulus processing. Specifically, several studies found a decrease in activation in the primary visual cortex to visual stimuli under high compared to low visual load^12,13,16,17^, but see Ref.^71^. Furthermore, activity in the lateral occipital cortex to visual distractors was reduced by an increase in auditory perceptual load^18^. In the auditory modality, activity to auditory stimuli was decreased under high compared to low load in the auditory cortex^72–74^. However, some studies did not find load effects in visual or auditory areas (e.g. Refs.^71,75^).

In the somatosensory modality, Dehghan Nayyeri et al.^44^ investigated effects of visual working memory load on brain activation to somatosensory distractors and did not find effects in SI or SII. *Load Theory*^2,76^ proposes a flexible locus of attention depending on the load of an ongoing task. During low load, information can be processed, while high load inhibits distractor processing during an early processing stage. Initially, the theory was limited to perceptual load, while later versions also included working memory load^77^. Remarkably, the theory suggested opposite phenomena for perceptual and working memory load^78–80^. However, this assumption was later revised^81^. Indeed, empirical work suggests similar effects of perceptual and working memory load on task-unrelated distractor processing (for review, see Ref.^5^). However, the comparative research on whether and how different task load manipulations affect sensory processing is still in its infancy, and there is a lack of theoretical models regarding the exact neurophysiological mechanisms of different task load approaches (see Ref.^5^).

Using a specific visual perceptual load task, we found no statistically significant effect of the load manipulation on somatosensory activation in the current study. This finding may suggest that, while spatial attention has been shown to affect brain responses to somatosensory stimuli in several studies^37,40–42^, load manipulations (during a given attentional focus) do not modulate activity, at least in certain crossmodal load paradigms. This finding might point to a difficulty in suppressing the processing of somatosensory distractors. One possible reason is the saliency and behavioural significance of the stimuli^82^. In the visual modality, it has been shown that distractor saliency affects their processing under load conditions^5^. Since somatosensory stimuli signal a possible danger for the body, these stimuli could be associated with stronger behavioural relevance than low-intensity visual stimuli. However, findings might also be associated with the specific experimental design, including its crossmodal nature and, therefore, separable attentional resources and other study features as discussed below.

While we did not find a statistically significant difference between load conditions despite a comparably large sample size, this absence of an effect might also represent a threshold phenomenon depending on the statistical procedures used. Based on Figs. 4 and 5, which seem to show a trend of differences between load conditions, this possibility cannot be excluded. Future studies with larger sample sizes and/or other analytical methods could perhaps detect statistically significant effects. Assuming that the clusters in 4 could serve as ROIs in future studies, in which betas can be averaged to avoid the problem of overly conservative thresholds in fMRI research, and that the observed differences between load conditions represent a reliable effect, it is possible to estimate the necessary sample sizes for future studies. Power calculations (G*Power 3.1.9^30^) yield required sample sizes for a significant paired t-test (α = 0.05) of 483 (SI, hand area), 211 (SI, operculum), 430 (SII, ipsilateral) and 1876 (SII, contralateral). Thus, even if we might have missed an effect, the required sample sizes for the most liberal way of analyzing the difference would be unusually large. Please also note that our exploratory Bayesian statistics for the mean beta differences between load conditions based on the data shown in Fig. 4 suggest anecdotal to moderate evidence for the null hypothesis. Thus, the additional exploratory analyses support the notion of the absence of differences between load conditions. Furthermore, our analysis of target trials shows that we could detect meaningful differences between conditions associated with perceptual load.

The current study used a crossmodal design: manipulating load in the visual modality while presenting somatosensory distractors. This procedure allowed an easy implementation of a perceptual load manipulation based on a previous visual study^20^. However, even though load effects are consistently shown in both unimodal and multimodal studies^5^, it has been suggested that differences between uni- and multimodal designs may exist, leading to stronger effects in unimodal designs (for discussion see Ref.^5^). At least in unimodal designs, perceptual load might be at least partially due to biased competition and lateral inhibition in early sensory areas (e.g. Ref.^11^). This would suggest a more likely sharing of attentional resources in unimodal than in crossmodal studies. Unimodal somatosensory studies or direct comparisons between unimodal and crossmodal studies could investigate this issue in more detail in future studies.

We would like to discuss some limitations of our study. We only used two load levels. Future studies could use a parametric range of load levels. Furthermore, we used a continuous load task in which responses to targets and the presentation of somatosensory stimuli were decorrelated. While this procedure allows investigating load effects independently from response-related effects, it might not represent the best approach to investigate the relevant timing of the interaction between load and distractors. The sustained design also made it difficult to investigate the perceptual load effect in visual areas. Thus, future studies may use different load tasks and timings between task features and distractor presentations. This would allow understanding better when, where and to what extent distractor processing can be inhibited by the perceptual load of a given task and how this relates to task-associated activations. This point, however, does not only concern the somatosensory modality, as highlighted in a recent review^5^. Another limitation arises from the fact that no measurement of somatosensory processing took place without a visual task to gain insight into effects of the visual task regardless of load on somatosensory processing. Future studies might use nested block/event-related designs with high-load blocks, low-load blocks, somatosensory attention blocks, and present and absent somatosensory stimuli. This would allow investigating baseline activations, sustained effects of perceptual load in visual and somatosensory cortex, and interactions with these sustained responses during the presentation of somatosensory stimuli.

Furthermore, we only investigated brain responses to somatosensory stimuli, which could be clearly perceived. Future studies should investigate also load effects on ERPs to weaker somatosensory stimuli with intensities around the detection threshold. Ideally, a parametric design with different load levels and different intensities of somatosensory stimulation would allow getting a detailed picture of possible load effects. Finally, the current study used a crossmodal load design. It remains an open question whether findings change in unimodal designs where earlier effects might be expected due to more strongly shared attentional resources^5^. Direct manipulations of unimodal and crossmodal load within one and the same study could investigate this issue in more detail in future studies.

## Conclusion

To conclude**,** we investigated the effect of a visual perceptual load manipulation on neural responses to somatosensory distractors in the primary and secondary somatosensory cortex. We found no significant effects on somatosensory activation for the utilized kind of crossmodal load. This lack of effects of at least some forms of visual perceptual load on somatosensory processing might be due to behavioural relevance of discrete somatosensory stimuli and separable attentional resources for the somatosensory and visual modality. However, we suggest that future studies should further investigate this issue using a variety of experimental approaches based on the current results and points raised in the discussion.

## Data Availability

The datasets analysed during the current study are available from the corresponding author on reasonable request.
